# Supplementary material for: Genetic study links components of the autonomous nervous system to heart-rate profile during exercise
Source: Nat Commun. 2018 Mar 1;9:898. doi: 10.1038/s41467-018-03395-6 (PMC5832790; doi:10.1038/s41467-018-03395-6)
Supplement: Supplementary file 1 — Supplementary Information [file 41467_2018_3395_MOESM1_ESM.pdf]

Supplementary Information

Genetic study links components of the autonomous nervous system to heart-rate profile during exercise

Verweij et al.

Description: Supplementary Figures and Tables

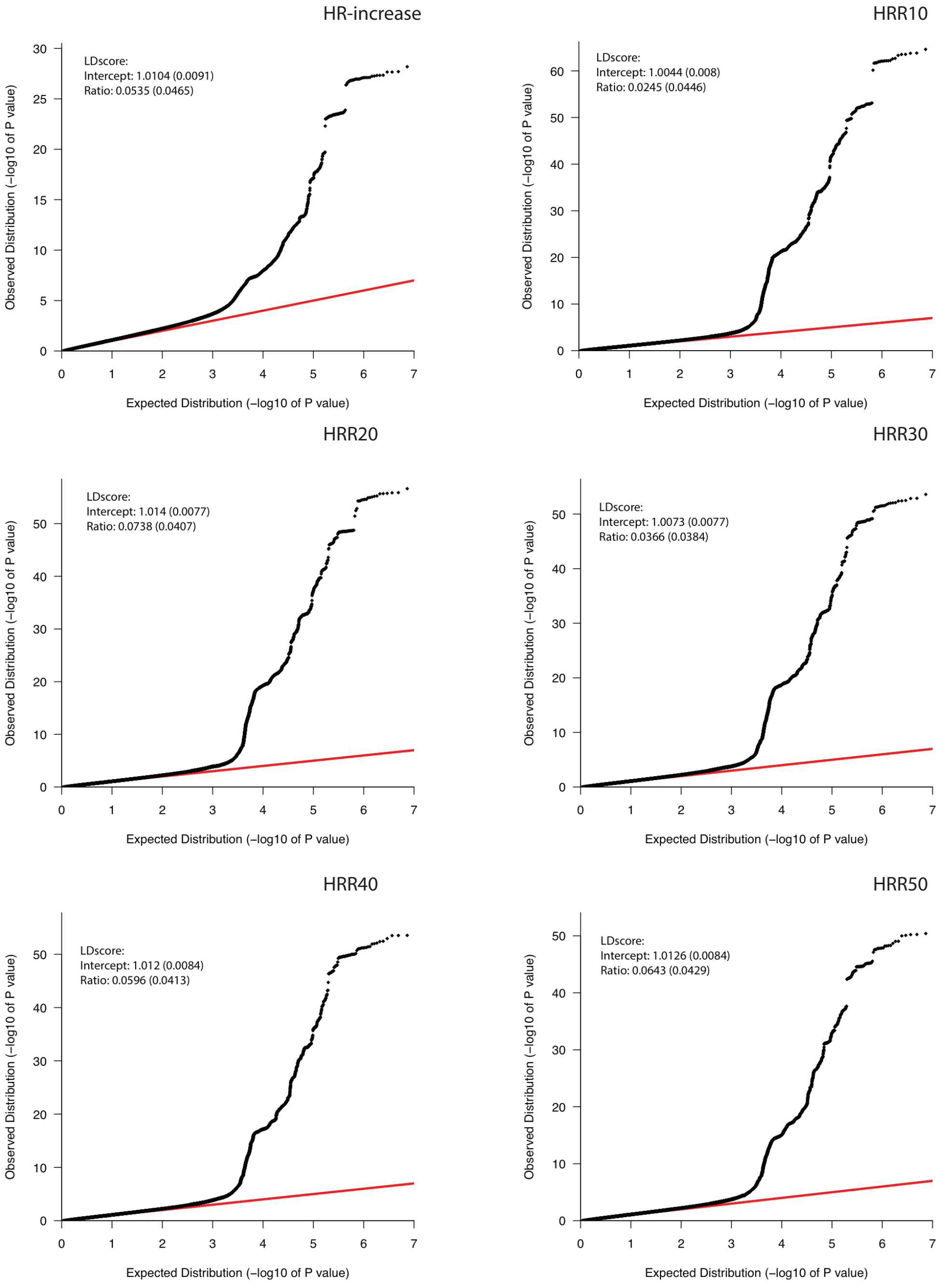

Supplementary Figure 1

The negative logarithm of the observed (y axis) and the expected (x axis) P value is shown for each SNP (black dots), and the red line indicates the null hypothesis of no true association. The intercept estimated by LD score[66]L suggest no deviation due to non-polygenic causes, the small residual deviation of ≤1% is most likely due to a mismatch between reference panel of 1000 Genomes used by LD score and UK Biobank (https://github.com/bulik/ldsc/wiki/Heritability-and-Genetic-Correlation) .

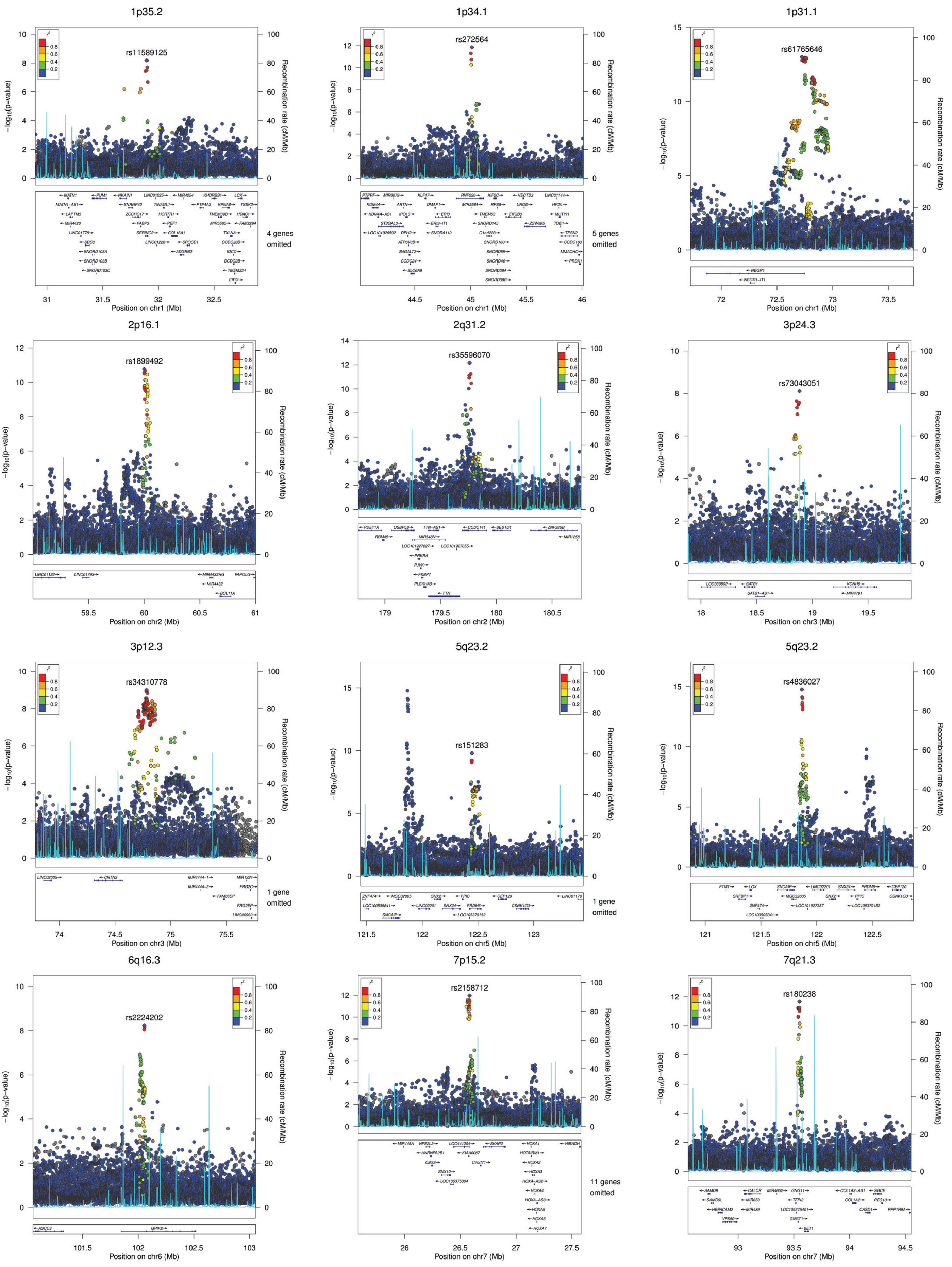

Supplementary Figure 2

The regional association plots for each of the identified loci. For each loci, the location of SNPs and genes and the direction of transcription are shown in the lower portion of the figure, the chromosomal position is shown on the x axis. The y axis on the left indicate the significance of association (−log10 P) for the SNPs that are colored according to the LD with the sentinel SNP; the light blue line indicates the recombination rate and corresponds with the right y-axes.

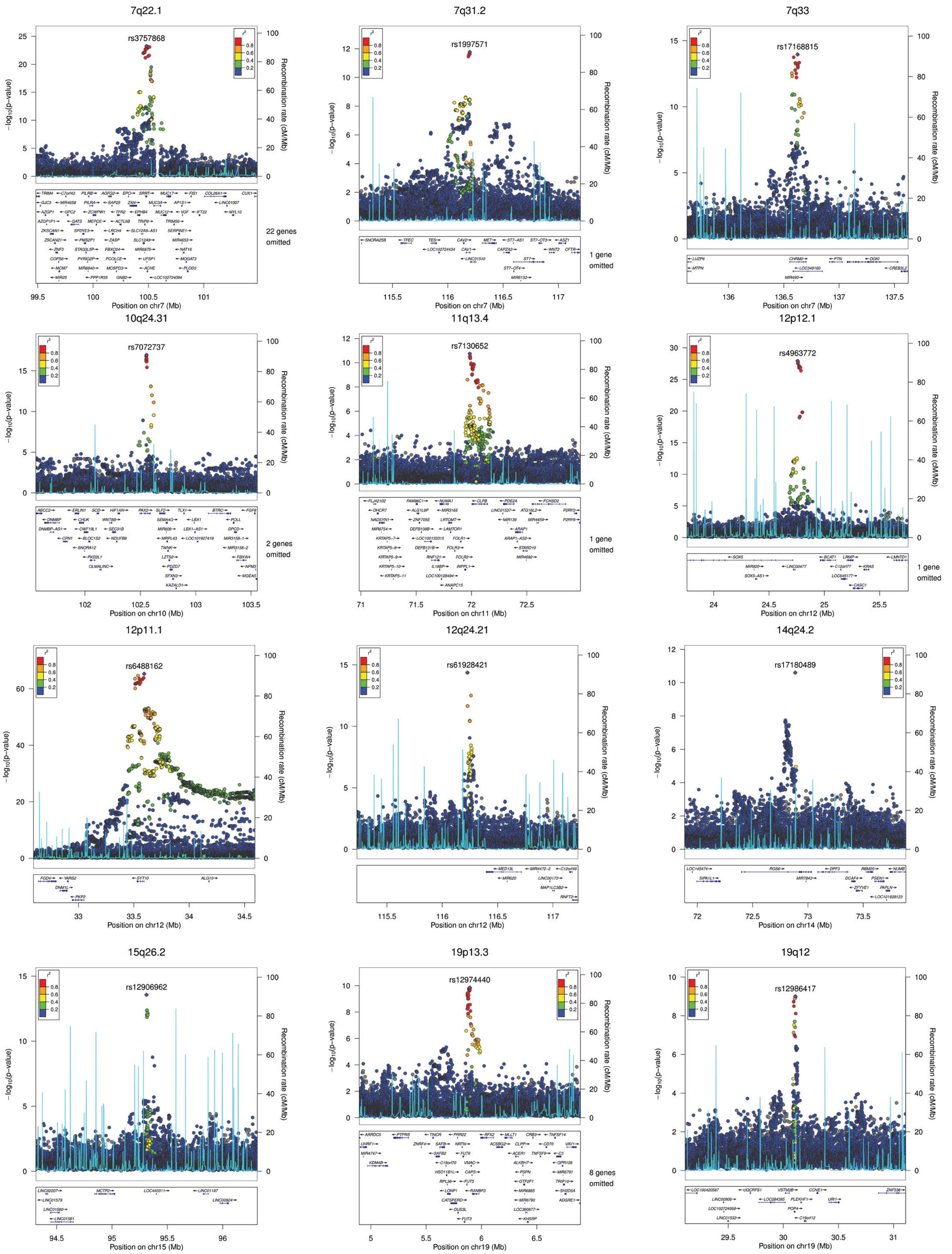

Supplementary Figure 2 (continued)

The regional association plots for each of the identified loci. For each loci, the location of SNPs and genes and the direction of transcription are shown in the lower portion of the figure, the chromosomal position is shown on the x axis. The y axis on the left indicate the significance of association (−log10 P) for the SNPs that are colored according to the LD with the sentinel SNP; the light blue line indicates the recombination rate and corresponds with the right y-axes.

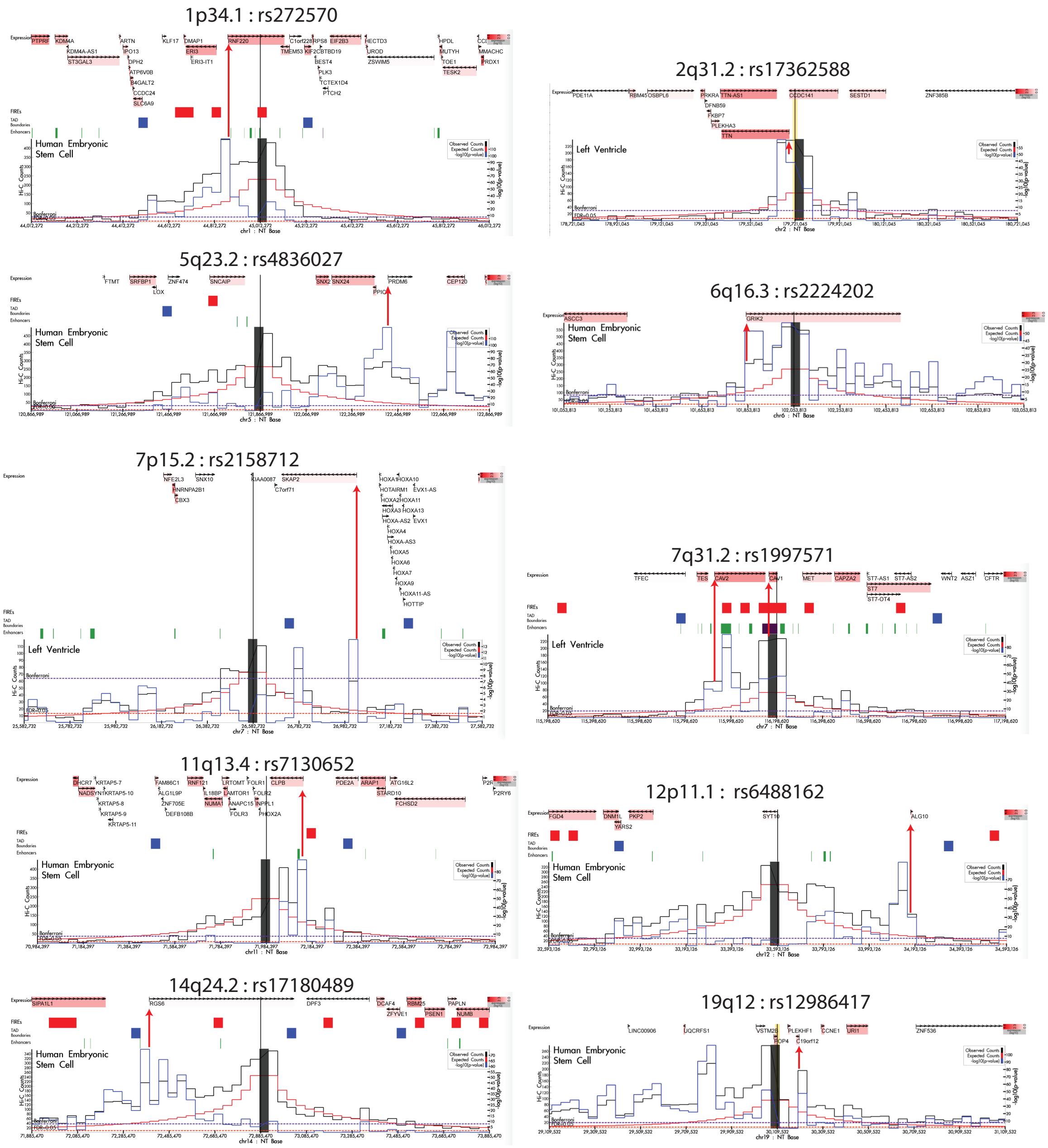

Supplementary Figure 3

Ten 4C plots are shown; depicting the long rang interactions by Hi-C data in the region of the sentinel SNPs and genes in the neighborhood (at the top of each figure). The black vertical line in the middle indicates the location of the sentinel SNP (the 'anchor' position); the black horizontal line indicates observed interaction counts, the red horizontal line expected interaction counts and the blue horizontal line indicates bonferonni significant interactions (threshold shown in purple dotted line). The red arrow indicates that the significant interaction is overlapping a promoter region of a candidate gene.

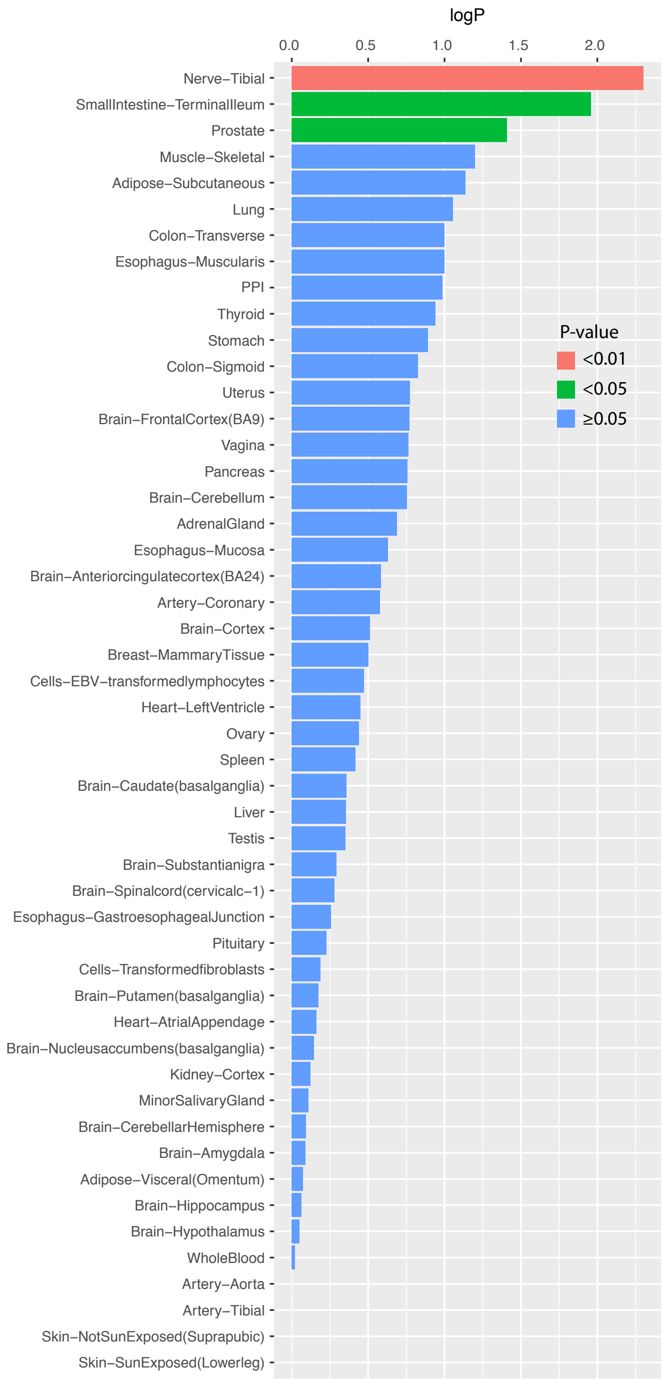

Supplementary Figure 4 Enrichment based on gene-expression in different tissues of GTEx for the 36 candidate genes using GLITTER (*Liu, Xiangtao, et al, Scientific reports 6 (2016))*. The x-axis is the –log (p-value) is a measure of the likelihood that the same or a greater number of connected gene pairs from random gene sets are observed, compared to the number of connected gene pairs among the 36 candidate genes. The y-axis labels the different tissues examined. The following settings were used: Pearson (correlation type), cut-off for connectedness (0.7), permuations (1000). Url: http://www.han-lab.org/GLITTER/default/index.

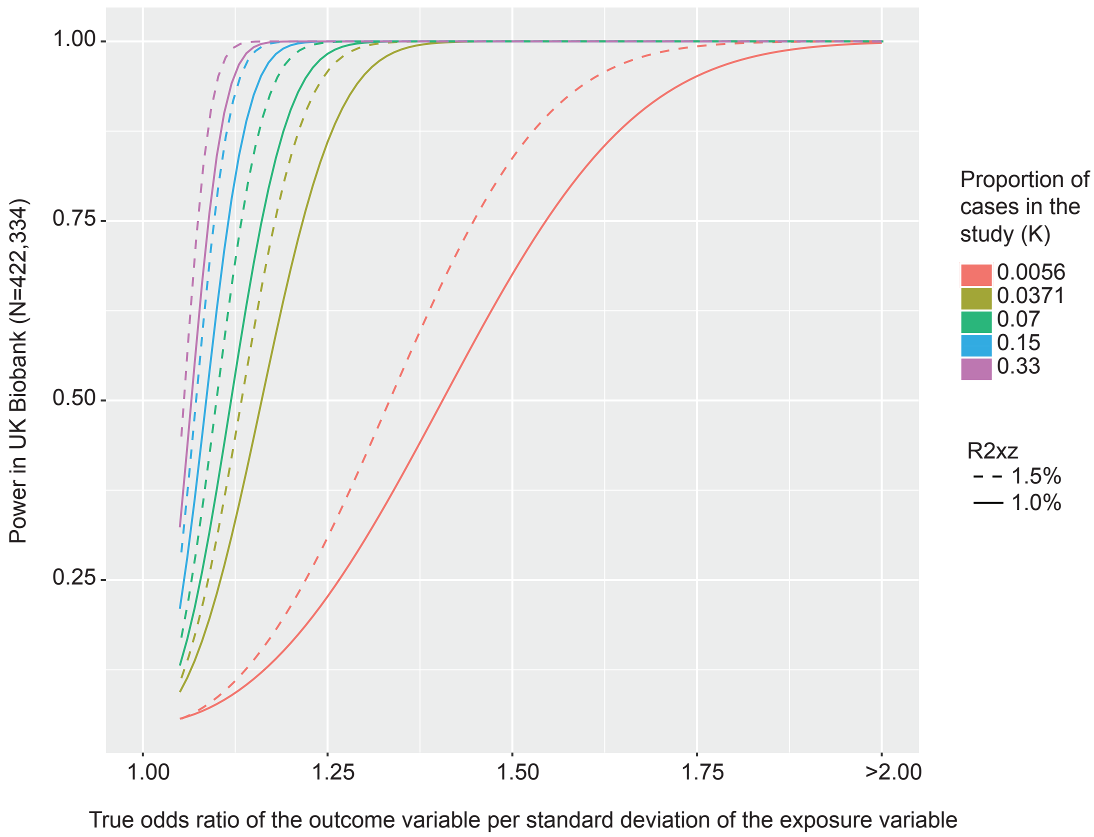

Supplementary Figure 5

Power calculations. The Fig. depicts the power for a range of odds ratio's, up to an odds ratio of 2 (100% power). R2xz is the proportion of variance explained for the association between the polygenic score and the exposure. The polygenic score explained 1.73% of the phenotypic variation, given that this was determined in the same dataset of the SNP-discovery, we hypothesize that the true value lies around 1% - 1.5% and based our power calculations on this. Values for K (case/control proportions) correspond to the disease phenotypes described in Table 2**.**

Supplementary Table 1. Baseline characteristics of UK Biobank participants that were included in the GWAS (N=58,818).

**Characteristic**		**Mean/Median (SD/IQR/%)**
Height(cm), mean (SD)		169.2 (9.2)
BMI(kg/m2), mean (SD)		27.1 (4.4)
Age (years), median (IQR)		58.3 (50.7, 63.6)
SDNN (seconds), median (IQR)		27.8(21.4-33.1)
RMSSD (seconds), median (IQR)		30.9 (24.7-37.8)
Resting heart rate, pre-exercise (bmp), mean (SD)		71.5 (11.7)
Heart rate recovery at 10s (bpm), mean (SD)		17.8 (7.7)
Heart rate recovery at 20s (bmp), mean (SD)		23.4 (9.1)
Heart rate recovery at 30s (bmp), mean (SD)		27.5 (9.8)
Heart rate recovery at 40s (bmp), mean (SD)		30.6 (10.4)
Heart rate recovery at 50s (bmp), mean (SD)		32.9 (10.8)
Heart rate-increase (bmp), mean (SD)		45.6 (12.9)
Exercise duration (seconds), median (IQR)		362.2 (361.9, 362.5)
Max Load during exercise, mean (SD)		83.4 (23.9)
Physical activity behavior categorized	Ideal	41425 (70.5%)
	Intermediate	13865 (23.6%)
	Poor	3436 (5.9%)
Diastolic blood pressure (mmHg)		83.5 (9.4)
Systolic blood pressure (mmHg)		134.9 (18.7)
Males (%)		28148 (47.9%)
Myocardial infarction (%)		843 (1.4%)
Cerebrovascular accident (%)		898 (1.5%)
Atrial fibrillation/Atrial flutter (%)		474 (0.8%)
Type 2 Diabetes (%)		1643 (2.8%)
Aortic valve disease (%)		96 (0.2%)
Cancer, malignant (%)		5574 (9.5%)
Self-reported Ethnicity	White	54137(92.04)
	Other/Unknown	1024(1.74)
	Asian	1744(2.97)
	Black	1445(2.46)
	Mixed	468(0.8)

Supplementary Table 2. Secondary signals. Secondary signals were initially identified using Plink's clumping procedure on LD (r2<0.01) and confirmed in a multivariate regression model that included both the primary snp and the potential secondary SNP, as shown in this table. This analysis confirmed two secondary signals at genome wide significance (in green), rs17362588 on chromosome 2 for HRR10 and rs4836027 on chromosome 5 for HRR50.

Phenotype	SNP	Chr	Pos	SNP adjusted for in the model	Beta	Se (robust)	t	P	Secondary/ Primary	Significant
HRR10	rs17362588	2	179721046	rs35596070	0.067	0.011	-6.31	2.79E-10	Secondary	1
HRR10	rs35596070	2	179759692	rs17362588	0.063	0.008	-7.65	2.10E-14	Primary	1
HRR50	rs4836027	5	121866990	rs151283	-0.040	0.006	-6.21	5.17E-10	Secondary	1
HRR50	rs151283	5	122446619	rs4836027	-0.042	0.007	-6.35	2.21E-10	Primary	1
HRR10	rs73106534	12	33502593	rs6488162	-0.179	0.048	-3.75	1.75E-04	Secondary	
HRR10	rs6488162	12	33593127	rs73106534	0.100	0.006	16.51	4.52E-61	Primary	1
HRR20	rs73106534	12	33502593	rs6488162	-0.188	0.047	-4.03	5.60E-05	Secondary	
HRR20	rs6488162	12	33593127	rs73106534	0.094	0.006	15.39	2.57E-53	Primary	1
HRR30	rs188900300	12	38641660	rs826878	-0.101	0.024	-4.23	2.32E-05	Secondary	
HRR30	rs826878	12	39088837	rs188900300	0.052	0.006	8.73	2.61E-18	Primary	1
HRR10	rs188900300	12	38641660	rs826878	-0.105	0.024	-4.47	7.87E-06	Secondary	
HRR10	rs826878	12	39088837	rs188900300	0.053	0.006	8.84	1.03E-18	Primary	1

Supplementary Table 3 Long range interacting genes (Hi-C) in left ventricle (LV), right ventricle (RV), human embryonic stem cell (H1) or neural progenitor cell (NPC). Please also see **Online Figure 3** which more accurately described the interactions.

SNP	Significant Long Range Hi-C	H1 (-log10(p-value)/counts/expected)	LV (-log10(p-value)/counts/expected)	NPC (-log10(p-value)/counts/expected)	RV (-log10(p-value)/counts/expected)
rs11589125		PEF1 HCRTR1 (1.02/108/94)	SDC3 (0.70/19/15)	TXLNA IQCC DCDC2B CCDC28B (0.43/8/6)	MATN1-AS1 MATN1 (0.31/5/4)
rs272564	RNF220	RNF220 (125.76/446/110)	RNF220 (21.03/106/35)	RNF220 (17.21/71/20)	RNF220 (8.25/40/13)
rs61765646		NEGR1-IT1 (13.48/109/48)	NEGR1-IT1 (2.56/31/17)	NEGR1-IT1 (2.87/23/11)	NEGR1-IT1 (0.40/8/6)
rs1899492		BCL11A (.99/114/31)	BCL11A (4.89/30/12)	BCL11A (5.34/24/8)	BCL11A (1.95/11/4)
rs17362588	TTN	TTN (51.59/532/253)	TTN (43.86/238/81)	CCDC141 (3.18/40/22)	TTN (8.57/66/29)
rs35596070	TTN	TTN (51.59/532/253)	TTN (43.86/238/81)	CCDC141 (3.18/40/22)	TTN (8.57/66/29)
rs73043051		SATB1 (12.34/135/67)	KCNH8 (5.25/56/29)	SATB1 (1.05/20/14)	KCNH8 (1.80/19/10)
rs34310778		CNTN3 (0.04/99/112)	CNTN3 (2.65/54/35)	CNTN3 (1.37/29/20)	CNTN3 (0.98/18/12)
rs4836027	PRDM6	PRDM6 (127.20/294/47)	CEP120 (10.70/41/11)	PRDM6 (18.73/53/11)	PRDM6 (3.71/18/6)
rs151283		PRDM6 (31.01/476/264)	SNCAIP (5.84/33/12)	PRDM6 (5.21/86/51)	PRDM6 (1.63/42/30)
rs2224202	GRIK2	GRIK2 (51.61/308/112)	GRIK2 (0.95/43/35)	GRIK2 (6.15/47/20)	GRIK2 (2.05/23/13)
rs2158712	SKAP2	HOXA3 HOXA4 HOXA-AS3 HOXA5 HOXA6 HOXA7 (5.13/66/36)	SKAP2 (14.21/60/18)	HOXA1 HOXA2 HOTAIRM1 HOXA-AS2 (1.16/15/9)	HOXA3 HOXA4 HOXA-AS3 HOXA5 HOXA6 HOXA7 (0.48/7/5)
rs180238		BET1 (95.85/502/167)	BET1 (65.07/226/55)	BET1 (4.81/60/33)	BET1 (8.85/53/20)
rs3757868		MUC3A (0.27/220/221)	GJC3 (0.83/13/9)	NAT16 VGF (0.68/18/14)	COL26A1 (0.53/8/6)
rs1997571	CAV2,CAV1	CAV2 (60.01/274/83)	CAV1 (47.27/228/72)	CAV2 (18.97/65/16)	CAV1 (7.21/58/26)
rs17168815		LUZP6 (12.43/61/20)	CHRM2 (6.75/98/55)	LUZP6 (3.42/16/5)	DGKI (0.45/5/3)
rs7072737		FAM178A (2.18/168/137)	ERLIN1 (0.51/16/13)	NDUFB8 NDUFB8 HIF1AN SEC31B (0.68/20/16)	WNT8B (0.17/8/9)
rs7130652	CLPB	CLPB (93.52/397/114)	CLPB (10.76/84/36)	CLPB (3.04/38/21)	CLPB (5.11/33/13)
rs4963772		KRAS (0.03/25/32)	BCAT1 (2.41/36/22)	BCAT1 (2.04/24/13)	BCAT1 (0.30/9/8)
rs6488162	ALG10	ALG10 (30.20/128/37)	ALG10 (2.39/26/14)	YARS2 (3.89/21/8)	YARS2 (1.39/10/5)
rs61928421		C12orf49 RNFT2 (0.67/26/21)	C12orf49 RNFT2 (2.05/18/9)	C12orf49 RNFT2 (0.07/4/6)	C12orf49 RNFT2 (0.46/5/3)
rs17180489	RGS6	RGS6 (75.45/215/43)	RGS6 (24.23/71/15)	RGS6 (4.23/25/10)	RGS6 (8.09/25/6)
rs12906962		LINC00924 (9.58/64/26)	LINC00924 (5.71/30/11)	LINC00924 (2.72/15/6)	LINC00924 (0.50/6/4)
rs12974440		RANBP3 (9.95/132/71)	EMR1 (3.04/12/3)	NRTN (2.26/28/16)	SAFB SAFB2 (1.07/9/5)
rs12986417	C19orf12	C19orf12 (47.40/195/55)	C19orf12 (15.45/69/21)	C19orf12 (4.00/28/12)	C19orf12 (5.71/26/8)
