# Supplementary material for: Genetic study links components of the autonomous nervous system to heart-rate profile during exercise
Source: Nat Commun. 2018 Mar 1;9:898. doi: 10.1038/s41467-018-03395-6 (PMC5832790; doi:10.1038/s41467-018-03395-6)
Supplement: Supplementary file 3 — Description of Additional Supplementary Files [file 41467_2018_3395_MOESM3_ESM.pdf]

Description of Additional Supplementary Files

File Name: Supplementary Data 1
Description: Genome wide association results (P<8.3×10-9) for all 6 traits.

File Name: Supplementary Data 2
Description: Post hoc sensitivity analyses.

File Name: Supplementary Data 3
Description: Coding variants in LD with the sentinel SNPs.

File Name: Supplementary Data 4
Description: eQTLs in LD with the sentinel SNPs.

File Name: Supplementary Data 5
Description: Results of pathway analyses.

File Name: Supplementary Data 6
Description: Intersection between the newly identified loci and previously reported heart rate loci.

File Name: Supplementary Data 7
Description: Intersection between the newly identified loci and any other previously reported GWAS loci.

File Name: Supplementary Data 8
Description: Lookup of the newly identified loci in GWAS summary results using phenomescanner.

File Name: Supplementary Data 9
Description: Associations of genome-wide significant SNPs unadjusted, and after adjustments of resting HR, HR-variability and HR-increase.

File Name: Supplementary Data 10
Description: Polygenic risk score analyses in the UK Biobank cohort.
